# Impact of Temperature and Time Interval Prior to Immature Testicular-Tissue Organotypic Culture on Cellular Niche

**DOI:** 10.1007/s43032-020-00396-z

**Published:** 2020-12-15

**Authors:** Sujith Raj Salian, Riddhi Kirit Pandya, Sindhura Lakshmi Koulmane Laxminarayana, Hanumantappa Krishnamurthy, Aswathi Cheredath, Prathima Tholeti, Shubhashree Uppangala, Guruprasad Kalthur, Subeer Majumdar, Stefan Schlatt, Satish Kumar Adiga

**Affiliations:** 1 Department of Clinical Embryology, Kasturba Medical College, Manipal, Manipal Academy of Higher Education, Manipal, 576104 India; 2 Department of Pathology, Kasturba Medical College, Manipal, Manipal Academy of Higher Education, Manipal, 576104 India; 3 National Centre for Biological Sciences, TIFR, Bengaluru, India; 4 National Institute of Animal Biotechnology, Hyderabad, India; 5 Centre of Reproductive Medicine and Andrology, Albert-Schweitzer Campus 11, 48149 Münster, Germany

**Keywords:** Immature testicular tissue, Prepubertal age, Organotypic culture, Holding temperature, Ultraprofound hypothermic temperature

## Abstract

Cryopreservation of immature-testicular-tissue (ITT) prior to gonadotoxic treatment, while experimental, is the only recommended option for fertility preservation in prepubertal boys. The handling and manipulation of ITT before cryopreservation could influence the functionality of cells during fertility restoration, which this study explored by evaluating cellular niche and quality of mouse ITT subjected to various temperatures and time durations in vitro. ITT from 6-day-old mice were handled at ultraprofound-hypothermic, profound-hypothermic, and mild-warm-ischemic temperatures for varying time periods prior to 14-day organotypic culture. Viability, functionality, synaptonemal complex and chromatin remodeling markers were assessed. Results have shown that cell viability, testosterone level, and in vitro proliferation ability did not change when ITT were held at ultraprofound-hypothermic-temperature up to 24 h, whereas cell viability was significantly reduced (*P* < 0.01), when held at profound-hypothermic-temperature for 24 h before culture. Further, cell viability and testosterone levels in cultured cells from profound-hypothermic group were comparable to corresponding ultraprofound-hypothermic group but with moderate reduction in postmeiotic cells (*P* < 0.01). In conclusion, holding ITT at ultraprofound-hypothermic-temperature is most suitable for organotypic culture, whereas short-term exposure at profound-hypothermic-temperature may compromise postmeiotic germ cell yield post in vitro culture. This data, albeit in mouse model, will have immense value in human prepubertal fertility restoration research.

**Supplementary Information:**

The online version contains supplementary material available at 10.1007/s43032-020-00396-z.

## Introduction

The prevalence of childhood cancer has increased significantly over the last decades, with the likelihood to be diagnosed with cancer being higher in boys than in girls [1]. This situation urges the need to develop strategies for safeguarding the fertility of childhood cancer patients due to the increased risk of treatment-induced infertility [2].

Cryopreservation of immature testicular tissue (ITT) prior to gonadotoxic treatment is an experimental procedure but the only recommended option for fertility preservation in prepubertal boys [3, 4]. The cryopreserved stem cells isolated from ITT can either be autotransplanted or matured in vitro to derive functionally competent spermatozoa [5–7]. In contrast to the in vivo approaches, in vitro approach eliminates the risk of reintroducing malignant cells [8].

Fertility preservation centers offering ITT banking are limited worldwide, which implies that the place where the testicular biopsy is performed might not be the same as the place of tissue banking. This could lead to longer transit time and risk of exposing ITT to suboptimal conditions such as warm temperature. Due to constant turnover of stem cells, prepubertal testis is more sensitive to cancer treatment than that of adults [9]. However, it is possible that the way the ITT is handled, transported, and maintained before cryopreservation may affect the functionality of the tissue.

If the retrieved ITT can be cryopreserved with minimal manipulation, the chance to recover optimum number of cells for fertility restoration techniques might increase [10]. Hence, earlier studies have addressed the effects of varying tissue size, storage temperatures, and storage periods in porcine model [10] and in human [11–14], to recommend the ideal conditions. No loss of cell viability and structural integrity was observed during the length of cooling [10], and proliferative potential was unaltered when cooled tissue was thawed and xenografted [15]. On the other hand, human ITT preservation at 4 °C for 3 days did not impair tissue integrity, Sertoli cell morphology, stem cell population, and the incidence of apoptosis [13]. In vitro maturation of human germ cells from ITT is being attempted as a promising alternative to in vivo approaches [11, 16–19], hence handling and manipulation of ITT may play an important role in maintaining the functionality of cells. This study is unique in understanding the cellular niche and quality of mouse ITT in response to manipulations such as holding at varying time periods and temperature prior to in vitro organotypic culture.

## Materials and Methods

### Animals, Ethical Clearance and Testicular Tissue Collection

All experiments and animal handling were conducted in accordance with the institutional guidelines for animal experimentation after obtaining prior approval from the Institutional Animal Ethics Committee (approval #IAEC/KMC/93/2013). A total of eighty-two, male prepubertal Swiss albino 6-day postpartum (dpp) mice were used in the study. Animals were sacrificed by cervical dislocation, and the testes were collected in alpha minimum essential medium (α-MEM + Glutamax; 32571-036; Gibco™, Grand Island, USA) containing 1% (v/v) penicillin-streptomycin (Pen-Strep; 15140-122; Gibco™) and 5-μg/mL Nystatin (Nys; N3503; Sigma-Aldrich, St. Louis, USA). Testes were made fat-free using fine needles, under the stereomicroscope, and later randomly distributed/categorized for either holding-phase or cultured directly (described below or as depicted in Supplementary fig. 1). Fresh testes, prior to holding phase and/or culture were evaluated for baseline validation.

### Holding Phase of Testes

The holding phase temperature for testes ex vivo was categorized as ultraprofound-hypothermic (~ 4 °C), profound-hypothermic (22–24 °C), and mild-warm-ischemic (34 °C) based on earlier recommendations [20]. For experimental setup, the excised 6 dpp testes were carefully transferred into a 1.5-mL Eppendorf tube, containing 1 mL of α-MEM + Glutamax media supplemented with 10% knock-out serum replacement (KSR; 10828-010; Gibco™), alongside Pen-Strep and Nys, using a sterile forceps (2 testes/tube). The tubes were placed in either (i) ultraprofound-hypothermic, i.e., in a cooling unit maintained at ~ 4 °C (ii) profound-hypothermic, i.e., in a vertical laminar flow maintained at 22 °C, and (iii) mild-warm-ischemic, i.e., in an incubator maintained at 34 °C.

The holding phase interval was scheduled as 6 h (corresponding to short range shipment) and 24 h (corresponding to long range shipment). This time points are selected based on our experience from human gonadal tissue cryopreservation in fertility preservation programs. Following the holding phase, the testes were released back into a falcon dish containing media preequilibrated at respective conditions. The testes were then processed by removing tunica albuginea and each testis was divided into eight equal pieces of 2–5 mm^3^. ITT were subjected to baseline validation of viability, functional characteristics, or for organotypic culture.

The ITT manipulation was performed at specific temperature corresponding to the holding temperature. Briefly, ITT held at ultraprofound-hypothermic condition were handled on frozen-ice pack, whereas profound-hypothermic, and mild-warm-ischemic groups were set on a stereomicroscope heating plate (Thermo-plate; Tokai Hit, Shizuoka, Japan) maintained at 22 °C and 34 °C, respectively. The total handling-phase time, including removal of tunica albuginea, cutting, tissue fixation for baseline validation, or transferring to agarose bed organotypic culture system was < 30 min.

### Organotypic ITT Culture

The equally cut testicular fragments (2–5 mm^3^ in size) were picked using sterile microforceps, and placed on a half-soaked agarose bed (at gas-liquid interphase) for organotypic testicular culture as explained earlier [7] with minor modifications. Briefly, 24 h prior to the culture, the agarose beds were prepared by mixing presterilized 0.7% low melting agarose (A9539; Sigma-Aldrich) with pre-warmed α-MEM + Glutamax medium in 1:1 ratio at 37 °C. The mixture was allowed to solidify in a petri dish at room temperature, under aseptic conditions. The solidified gel was later cut into circular cylindrical beds which were then soaked overnight in α-MEM + Glutamax medium, supplemented with KSR (10%), antimicrobial agents (as stated above) and 0.3-μg/mL retinoic acid (R2625; Sigma-Aldrich). Prior to setup of the culture, the equilibrated beds were transferred into a 12-well culture dish (3512; Corning, USA), one bed per well, containing 500 μL of the culture medium. ITT fragment was placed on top of the half-soaked bed using a sterile forceps. ITT were cultured at 34 °C and 5% CO_2_ for 14 or 35 days, and the culture medium was completely replaced by fresh medium on the seventh day of the culture.

### Evaluation of ITT Ex Vivo

Cultures were monitored at regular intervals for microbial contamination. At the end of 14- or 35-day ex vivo culture, ITT were imaged using cellSens imaging software (Olympus, Japan) at × 4 magnification. Cultured ITT were assessed for structural, functional, and genetic parameters.

### Testicular Histology

Cultured ITT were fixed in Bouin’s solution for histological analysis. Excessive fixative was removed by washing in 50% and 70% alcohol for 15 min each. The fixed tissues were then manually processed for dehydration by acetone, clearing by xylene followed by infiltration, and impregnation by paraplast wax. The tissue was then embedded in paraplast wax and cut into 5-μm sections on slides. After deparaffinization and rehydration, the sections were stained with Hematoxylin and Eosin (H&E) and examined for the integrity of the tubules and cells.

### Quantification of Testicular Cells by DNA Flow Cytometry

The PI staining of testicular cells was similar to the protocol described by Bose et al. [21]. Briefly, an aliquot of ethanol-fixed testicular cells was washed with 1X HBSS and treated with 0.5% pepsin solution for 2 min at 37 °C. After centrifugation, cells were stained with PI staining solution (25 μg/ml PI, 40 μg/ml RNase, and 0.03% Nonidet P-40 in HBSS) at room temperature for 15 min. The PI-stained cells were analyzed on FACS Verse (Becton Dickinson, USA). The PI stained cells were excited at 488 nm, and the emitted fluorescence signals were collected at 586/42 nm band pass filter.

### Viability Assessment

**Fig. 1:**
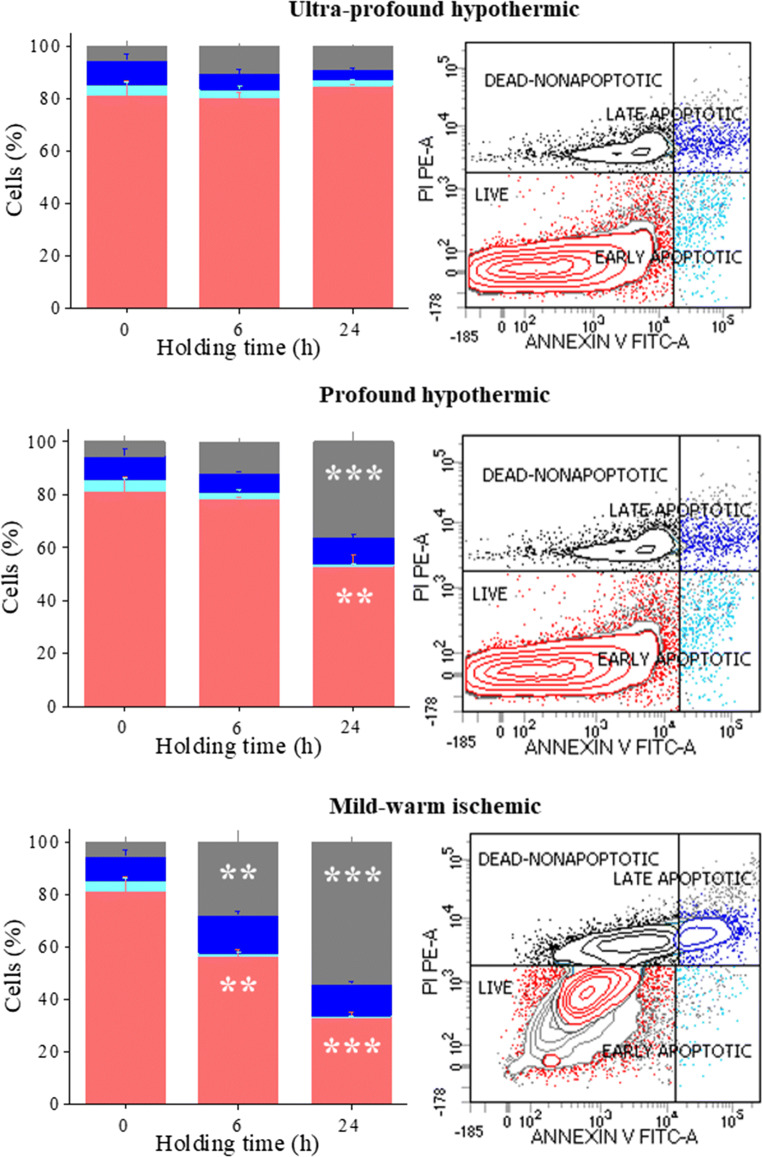
Flow cytometric analysis of ITT cells. Effect of varying holding time and temperature on ITT cell viability using Annexin-V/ propidium iodide (AV/PI) staining by flow cytometer. Flow cytometric analysis of ITT held at ultra-profound-hypothermic (top panel), profound-hypothermic and (middle panel), mild-warm-ischemic (lower panel) for 0, 6, and 24 h interval is shown in right hand panel, and, a representative cytogram of 24 h, in left hand panel. The data is represented in percentage, of live (red), early apoptotic (aqua), late apoptotic (blue), and dead (gray) population. **P < 0.01 and ***P < 0.001 vs. 0 h (control), *N* = 3 trial

**Fig. 2:**
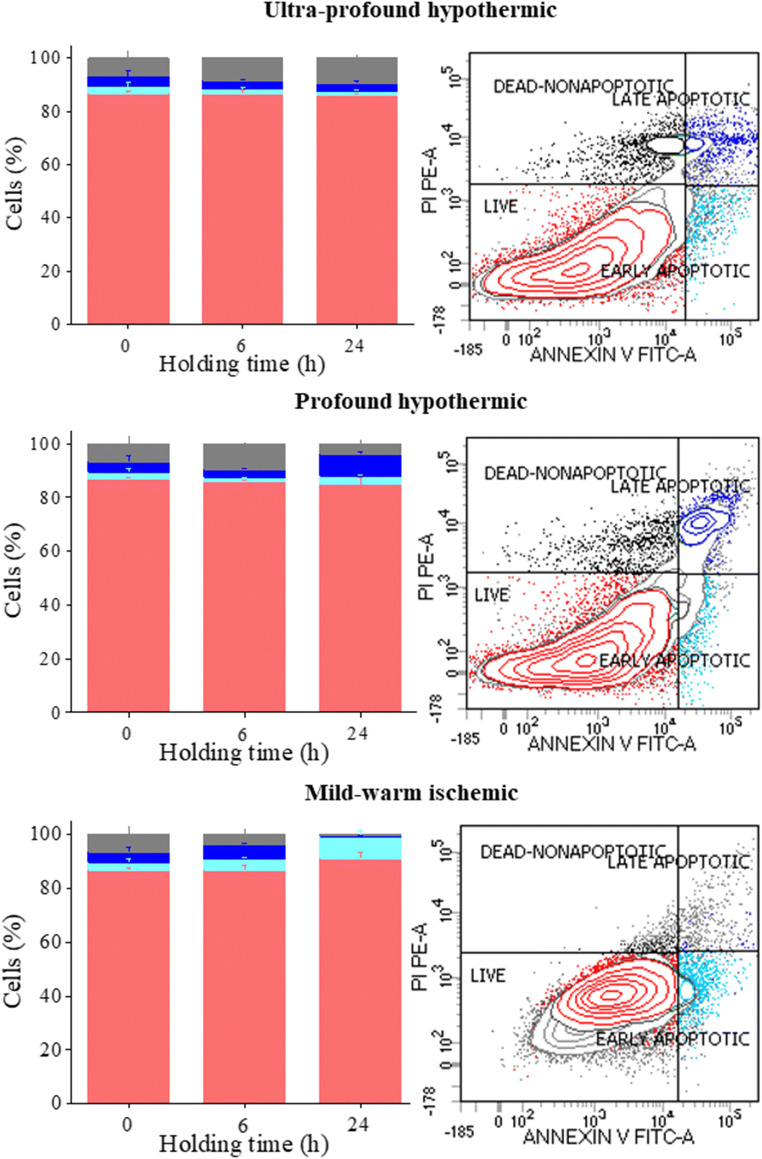
Flow cytometric analysis of in vitro cultured ITT cells. Effect of varying holding time and temperature on ITT cell viability after 14 days of in vitro culture using Annexin-V/ propidium iodide (AV/PI) staining by flow cytometer. Flow cytometric analysis of ITT held at ultraprofound-hypothermic (top panel), profound-hypothermic and (middle panel), mild-warm-ischemic (lower panel) for 0, 6 and 24 h interval is shown in right hand panel, and, a representative cytogram of 24 h, in left hand panel. The data is represented in percentage, of live (red), early apoptotic (aqua), late apoptotic (blue), and dead (gray) population. No statistical significance was found between the groups, *N* = 3 trial

Fluorescein isothiocyanate (FITC) Annexin-V/propidium iodide (PI) dead cell apoptosis kit (V13242, Invitrogen, USA) was used to detect cell viability according to the manufacturer’s instructions with minor modifications. Briefly, ITT were treated with trypsin-collagenase mixture (1:1) in α-MEM + Glutamax medium at 37 °C for 30 min and then mechanically dispersed using pipette. The suspension was passed through a nylon mesh into a sterile tube containing equal volume of α-MEM + Glutamax medium supplemented with 20% fetal calf serum. Single cell suspension was washed twice with medium followed by washing in cold phosphate buffered saline (PBS) at 110*g* for 8 min. The resultant pellet was resuspended in 100 μL of 1X Annexin-binding buffer, 5 μL of FITC Annexin-V and 1 μL PI (100 μg/mL) and incubated for 15 min at room temperature in dark. Following this, 400 μL of 1X Annexin-binding buffer was added with a target of one million cells per tube and mixed gently. An unstained control was run with each batch to identify autofluorescence. A fresh sample control at room temperature without incubation or culture was run to determine the live cell population. Flow cytometric analysis was performed on BD FACS CANTO II (Becton Dickinson Biosciences, USA). The cells stained with Annexin-V FITC and PI was excited using 488 nm laser. Debris and doublets were eliminated from the analysis using light scatter characteristics. Fluorescence signals of FITC were collected using 530/30 BP and PI using 585/42 BP. The early apoptotic cells bind only Annexin-V and late apoptotic cells bind both Annexin-V and PI. The double negative cells are considered as live cells. Biexponential scale was applied for the data analysis (Figs. 1 and 2). The experiments are repeated in triplicates (*N* = 3) for the reproducibility and statistical evaluation.

### Testosterone Measurement

Testosterone levels in ITT were measured using Demeditec testosterone ELISA kit (DE1559, Kiel, Germany), according to the manufacturer’s instructions with minor modifications. Briefly, ITT was homogenized in 1 mL of PBS followed by centrifugation at 2700*g* for 10 min, and the testosterone levels were measured in the cell lysate. Precoated wells were loaded with 25 μL of cell lysate and 200 μL of enzyme conjugate, mixed thoroughly and incubated for 60 min at room temperature. Wells were washed with wash buffer and incubated in 200 μL of substrate solution for 15 min. The reaction was terminated using stop solution and optical density was recorded at 450 nm using Multiskan™ FC Microplate Photometer (Thermo fisher scientific, Massachusetts, USA).

### Isolation of Total RNA and Reverse Transcription

Total RNA was extracted from ITT using TRIzol reagent (15596018, Ambion life technologies, USA). Total RNA (1 μg) was reverse transcribed using random primers by high capacity cDNA RT kit (4368814, Applied biosystems, USA) according to manufacturer’s protocol.

### Gene Expression Analysis

Quantitative polymerase chain reaction (qPCR) was carried out using Premix Ex Taq kit (RR390A, TaKaRa Bio, Japan), in StepOne™ Real-Time PCR System (Thermo Fisher Scientific, USA). TaqMan assay (Thermo fisher scientific, USA) for postmeiotic markers (*Sycp1* and *Sycp3*) and chromatin remodeling proteins (*Tnp2* and *Prm1*) were used (Supplementary Table 1). qPCR results were normalized to *Actb* and *Gapdh* housekeeping genes according to ΔCT methodology.

### Statistical Evaluation

The statistical significance level of mean ± SEM for the variables following normal distribution was calculated using one-way analysis of variance (ANOVA) or Kruskal–Wallis test if failed normality test (followed by Dunn’s test) by GraphPad InStat 3.0 statistical package (GraphPad Inc., USA). The graphs were plotted using Origin 8.0 (Origin Lab Corporation, USA).

## Results

### Optimization of Length of the Organotypic Testicular Tissue Culture

Five quantifiable populations in ITT, subjected to ultraprofound-hypothermic, profound-hypothermic and mild-warm ischemic handling conditions, based on the DNA content were determined by DNA flow cytometry [22]. These include (i) elongated spermatids (HC; H, hypostainability of elongated spermatids due to condensation of nuclear DNA during spermiogenesis); (ii) round spermatids (1C); (iii) spermatogonia and testicular somatic cells (2C); (iv) spermatogonial cells synthesizing DNA (S-phase), and (v) primary spermatocytes and G2 spermatogonia (4C). It may be noted that the percentage of testicular somatic cells (Sertoli, Leydig, and peritubular myoid cells) is less than 3% of total testicular cells in adult mice [23] and falls within the 2C population.

Prior to the holding phase, the fresh ITTs were manipulated/handled in ultraprofound-hypothermic, profound-hypothermic-temperature, and mild-warm-ischemic temperature, were cultured up to 14 and/or 35 days. At the end of 14 day culture, the HC and 1C populations were approximately 11-fold higher (*P* < 0.001) in the ultraprofound-hypothermic group, while compensatory decrease in 2C population was observed (*P* < 0.001) and 4C population increased by 2-fold (*P* < 0.05) compared to 6 dpp ITT (fresh, not subjected to in vitro culture) (Table 1). Interestingly, the percentage of subpopulations was comparable between 14 and 35 days of in vitro culture, except a significant decrease in 1C population was observed in 35 day group (*P* < 0.01). Based on this observation, in vitro culture was restricted to only 14 days for subsequent experiments.

**Table 1:** Flow cytometric analysis of cell types from ITT (0 h holding; fresh tissues) subjected to varying holding conditions, prior to organotypic culture

Holding condition	Length of the culture (Days)	*N*	Percent distribution of cell types (mean ± SEM)				
			HC	1C	2C	S-phase	4C
Ultra-profound hypothermic	0	5	1.3 ± 0.2	1.5 ± 0.5	76.0 ± 1.9	5.8 ± 0.7	9.4 ± 0.6
	14	8	14.7 ± 1.9 ^c^	17.3 ± 1.9 ^c^	34.7 ± 2.7 ^c^	6.4 ± 0.5	17.0 ± 2.0 ^a^
	35	9	14.9 ± 1.4 ^c^	11.8 ± 1.6 ^b^	40.4 ± 3.4 ^c^	8.0 ± 0.5 ^a^	16.2 ± 1.6 ^a^
Profound hypothermic	0	6	2.0 ± 0.3	3.9 ± 1.1	67.6 ± 2.6	6.7 ± 0.3	10.2 ± 1.2
	14	8	13.5 ± 0.6 ^c^	17.5 ± 0.6 ^c^	37.6 ± 0.7 ^c^	5.6 ± 0.5	15.9 ± 0.8
	35	8	13.7 ± 1.6 ^c^	8.8 ± 1.1 ^b^	41.6 ± 0.9 ^c^	8.4 ± 0.7	20.8 ± 2.2 ^c^
Mild-warm-ischemic	0	5	2.7 ± 0.5	2.4 ± 1.1	75.5 ± 2.6	5.0 ± 0.6	9.9 ± 0.7
	14	7	12.2 ± 1.0 ^c^	16.1 ± 1.2 ^c^	37.1 ± 0.8 ^c^	5.9 ± 0.4	18.9 ± 1.7 ^b^
	35	7	14.5 ± 2.0 ^c^	9.5 ± 1.4 ^b^	40.4 ± 1.6 ^c^	7.8 ± 0.5 ^a^	19.9 ± 2.6 ^b^

^a^*P* < 0.05; ^b^*P* < 0.01; ^c^*P* < 0.001 vs. 0 day (6 dpp, fresh ITT) culture

### Manipulation of ITT Ex Vivo Affects the Cell Viability

ITT subjected to varying holding time and temperature was assessed for the cell viability using Annexin-V/ propidium iodide (AV/PI) staining. The flow cytometric measurement of AV corresponding signal provides a very sensitive method for detecting cellular apoptosis, while PI is used to detect nonviable population characterized by the loss of the integrity of the plasma and nuclear membranes (Figs. 1 and 2).

Holding ITT at ultraprofound-hypothermic-temperature up to 24 h did not impair cell viability (Fig. 1, top panel). In contrast, the number of live cells in profound-hypothermic-temperature group reduced significantly by the end of 24 h. Approximately 30% of the cells became nonviable (*P* < 0.01), which was accounted for dead cells population (*P* < 0.001). The early apoptotic and the late apoptotic cell number did not vary significantly across the groups similar to the ultraprofound-hypothermic group (Fig. 1, middle panel).

At mild-warm-ischemic holding condition, approximately 1.4- and 2.5-fold reduction in the number of live cells was observed at 6 h (*P* < 0.01), and at the end of 24 h, respectively, where only 33.4 ± 1.8% of cells were viable (*P* < 0.001). Conversely, the dead cell population increased by 10.4-fold at 6 h (*P* < 0.01), and 16.7-fold at 24 h (*P* < 0.001). On the other hand, the number of early and late apoptotic cells did not vary significantly across the groups (Fig. 1, lower panel).

ITT cultured in vitro for 14 days were subjected to AV/PI staining. Holding at ultraprofound-hypothermic-temperature for varying time periods followed by in vitro culture did not alter the number of live, dead, and apoptotic cell populations across the groups (Fig. 2, upper panel). Similarly, cell viability when held at profound-hypothermic temperature for 6 h was comparable to 0 h. However, a nonsignificant increase in the number of late apoptotic cells and increase in the number of dead cells were observed at 24 h holding time when compared to 0 h (Fig. 2, middle panel). Interestingly, holding at mild-warm-ischemic temperature for a period of 6 or 24 h prior to the 14 day culture did not affect the cell viability. Holding the tissue for 24 h resulted in a nonsignificant reduction in late apoptotic and dead cell population compared to 0 and 6 h (Fig. 2, lower panel).

### Altered Testosterone Levels in Mild-Warm-Ischemic Temperature

**Fig. 3:**
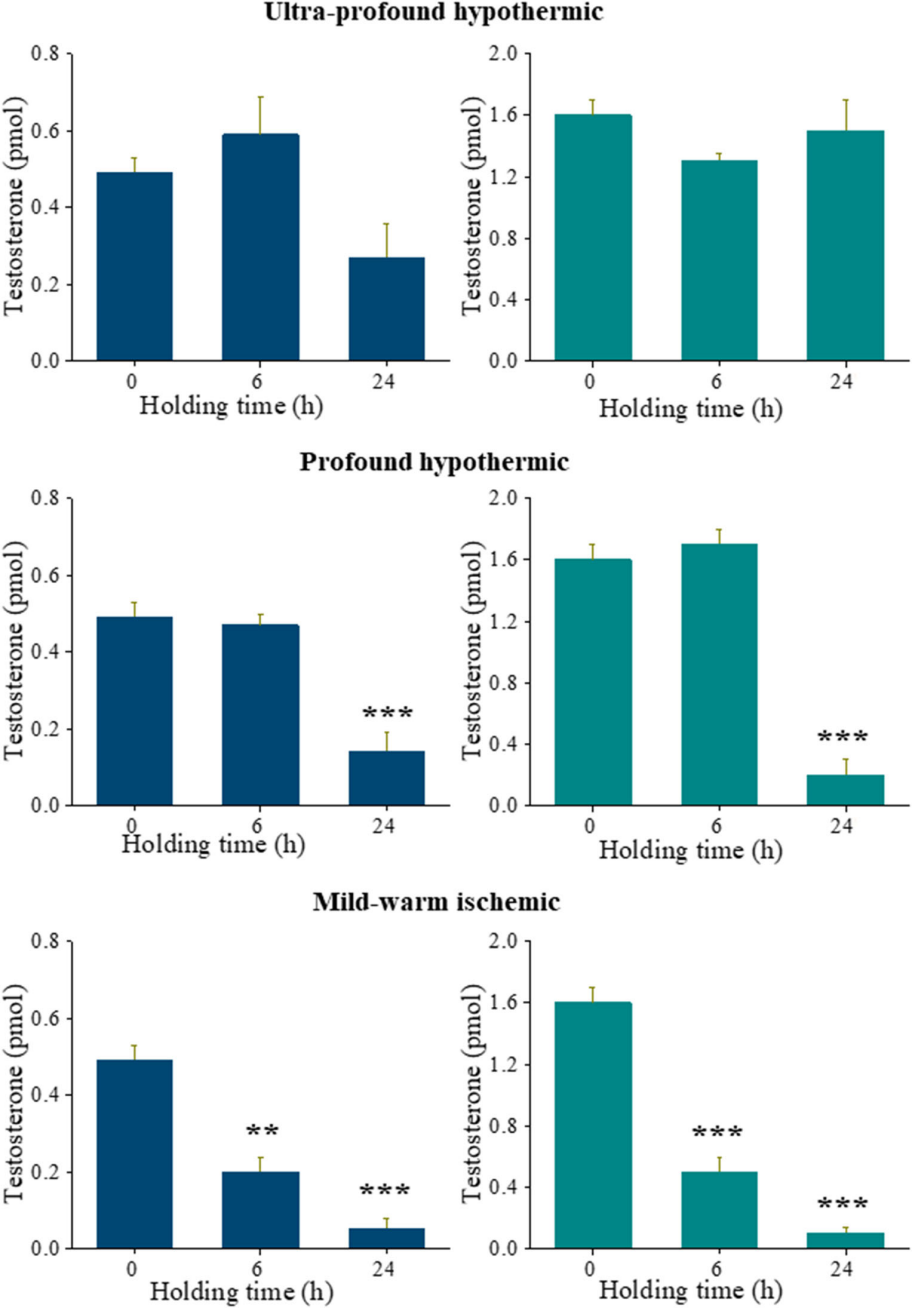
Testosterone measurement in ITT. Effect of varying holding time and temperature on levels of testosterone in ITT before and after 14 days of in vitro culture assessed using enzyme-linked immunosorbent assay (ELISA). The levels of testosterone in 6 dpp mice testes at 0 h interval was considered as control, in comparison to 6 and 24 h held ITT. The ITT were held at ultra-profound-hypothermic (upper panel) profound-hypothermic (middle panel) and mild-warm-ischemic (lower panel) temperatures. The testosterone levels were estimated before (left hand panel) and after (right hand panel) 14 day in vitro culture. The data is represented in mean ± SEM, ^**^P < 0.01 and ^***^P < 0.001 vs. 0 h (control), *N* = 3 trial

Testosterone levels, indicating the functionality of Leydig cells, were estimated at varying time intervals from ITT before and after in vitro culture. ITT subjected to holding at ultraprofound-hypothermic-temperature for 24 h showed a nonsignificant decline in testosterone level compared to other two groups (Fig. 3, upper left). However, at the end of 14-day in vitro culture, testosterone level was comparable with other groups (Fig. 3, upper right). On the other hand, a significant decline in testosterone level was found when ITT were held at profound-hypothermic-temperature for 24 h before (*P* < 0.001) (Fig. 3, middle left) and after 14 days of in vitro culture (*P* < 0.001) (Fig. 3, middle right). Interestingly, holding at mild-warm-ischemic-temperature for 6 h before culture has affected Leydig cells’ functionality significantly as testosterone was maintained at low levels before (*P* < 0.01) (Fig. 3, lower left) and after in vitro culture (*P* < 0.001) (Fig. 3, lower right).

### Morphology and Tissue Integrity, Post Organotypic Testicular Culture

**Fig. 4:**
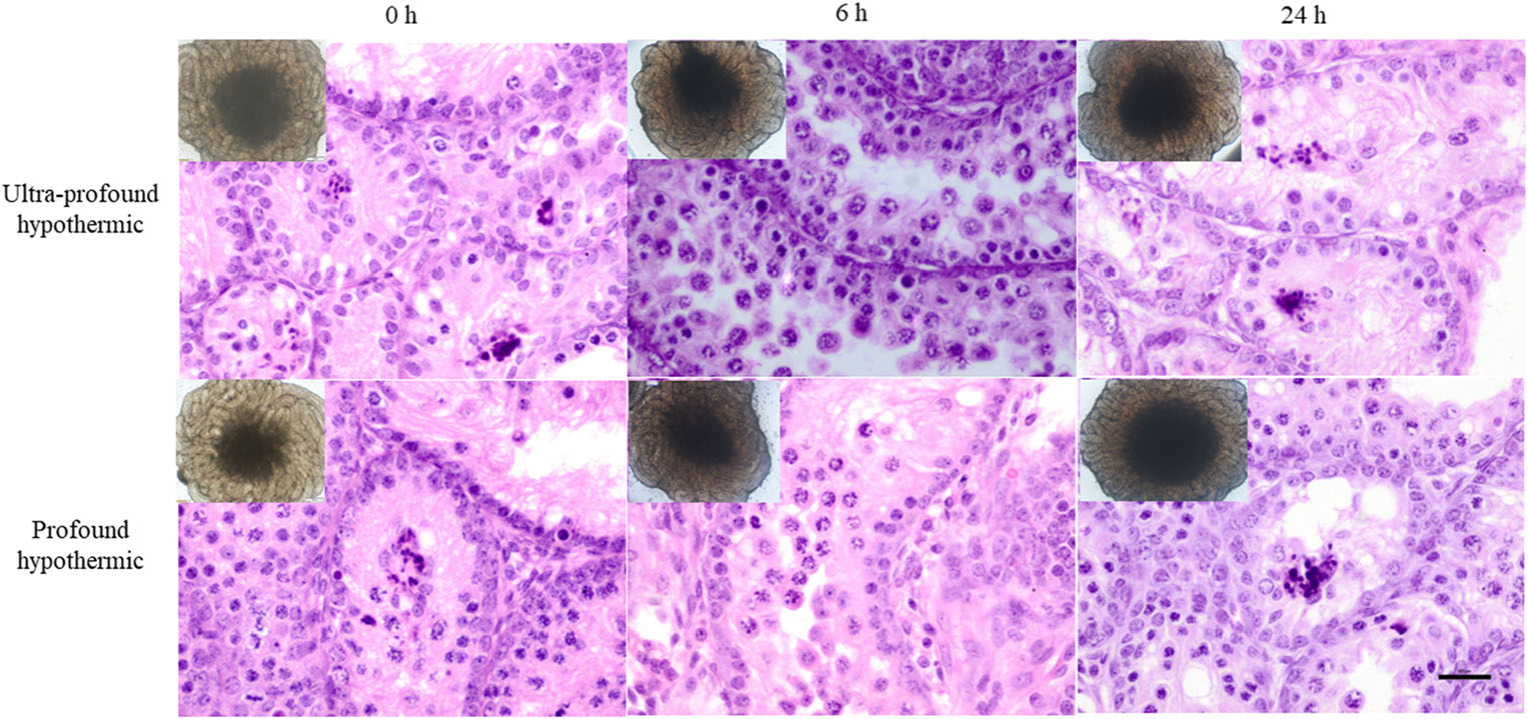
Histological analysis of cultured ITT. Effect of varying holding time and temperature on ITT held at ultraprofound-hypothermic and profound-hypothermic-temperature for 0, 6, and 24 h time periods on tissue morphology and appearance (inset) after 14 days of in vitro culture, as assessed under inverted microscope and imaged using CellSens imaging software (Olympus, Japan) at 4X magnification. Representative histological sections after H&E staining from corresponding ITT were imaged at × 40 magnification

The ITT subjected to organotypic testicular culture were monitored throughout the culture period. The ITT from ultraprofound and profound-hypothermic-temperature appeared healthy and expanded in shape, size, color, texture, and integrity of the tubules though central necrosis was evident in all the groups tested (Fig. 4, inset). Importantly, the ITT from the mild-warm-ischemic temperature were found shrunk in size, change in color, and texture (Supplementary fig. 2). However, qualitative assessment of tubules from ultraprofound and profound-hypothermic temperature has demonstrated marginal variations in structure and number of cells within the tubules (Fig. 4).

### Quantification of Cultured Testicular Cells

**Fig. 5:**
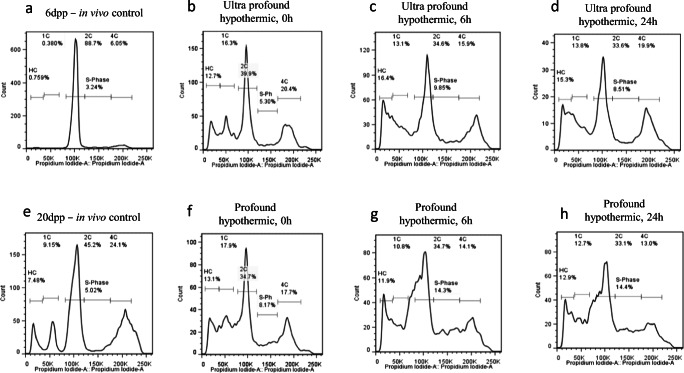
Analysis of testicular population postorganotypic culture. Representative flow cytogram depicting cells in various stages of cell cycle, 14 days after organotypic culture from ITT held at ultra-profound-hypothermic and profound-hypothermic-temperature for 0 (**b**, **f**), 6 (**c**, **g**), and 24 h (**d**, **h**) time periods. Age matched (6 and 20 dpp) in vivo control are shown in (**a**) and (**e**) respectively

In the next set of experiments, ITT subjected to holding phase for varying time intervals (6 and 24 h) in ultraprofound-hypothermic and profound-hypothermic-temperature were cultured up to 14 days. S-phase population was significantly higher at 6 and 24 h holding time in both ultraprofound-hypothermic and profound-hypothermic groups compared to 0 h (*P* < 0.01). Similarly, 1C population in 6 h holding time at profound-hypothermic-temperature was significantly lower compared to 0 h (*P* < 0.01). However, no significant changes were observed between other groups (Table 2; Fig. 5).

**Table 2:** Flow cytometric analysis of cell types from ITT on day 14 of organotypic culture, subjected to varying holding conditions and time periods

Holding condition	Holding time (h)	*N*	Percent distribution of cell types (mean ± SEM)				
			HC	1C	2C	S-phase	4C
Ultraprofound hypothermic	0	8	14.7 ± 1.9	17.3 ± 1.9	34.7 ± 2.7	6.4 ± 0.5	17.0 ± 2.0
	6	6	16.9 ± 2.4	16.0 ± 3.4	31.1 ± 3.6	10.6 ± 0.9 ^b^	12.9 ± 2.6
	24	6	12.3 ± 0.6	12.4 ± 0.9	35.3 ± 1.4	12.0 ± 0.8 ^c^	14.2 ± 1.0
Profound hypothermic	0	8	13.5 ± 0.6	17.5 ± 0.6	37.6 ± 0.7	5.6 ± 0.5	15.9 ± 0.8
	6	6	12.2 ± 1.5	11.0 ± 1.3 ^b^	36.3 ± 2.0	11.6 ± 1.1 ^b^	17.0 ± 1.3
	24	6	16.5 ± 1.2	14.1 ± 1.4	31.7 ± 2.4	11.5 ± 1.4 ^b^	15.0 ± 1.8

^a^*P* < 0.05; ^b^*P* < 0.01; ^c^*P* < 0.001 vs. 0 h (6 dpp, cultured for 14 days) ITT

### Expression of Sycp1 and Sycp3 Genes in ITT

**Fig. 6:**
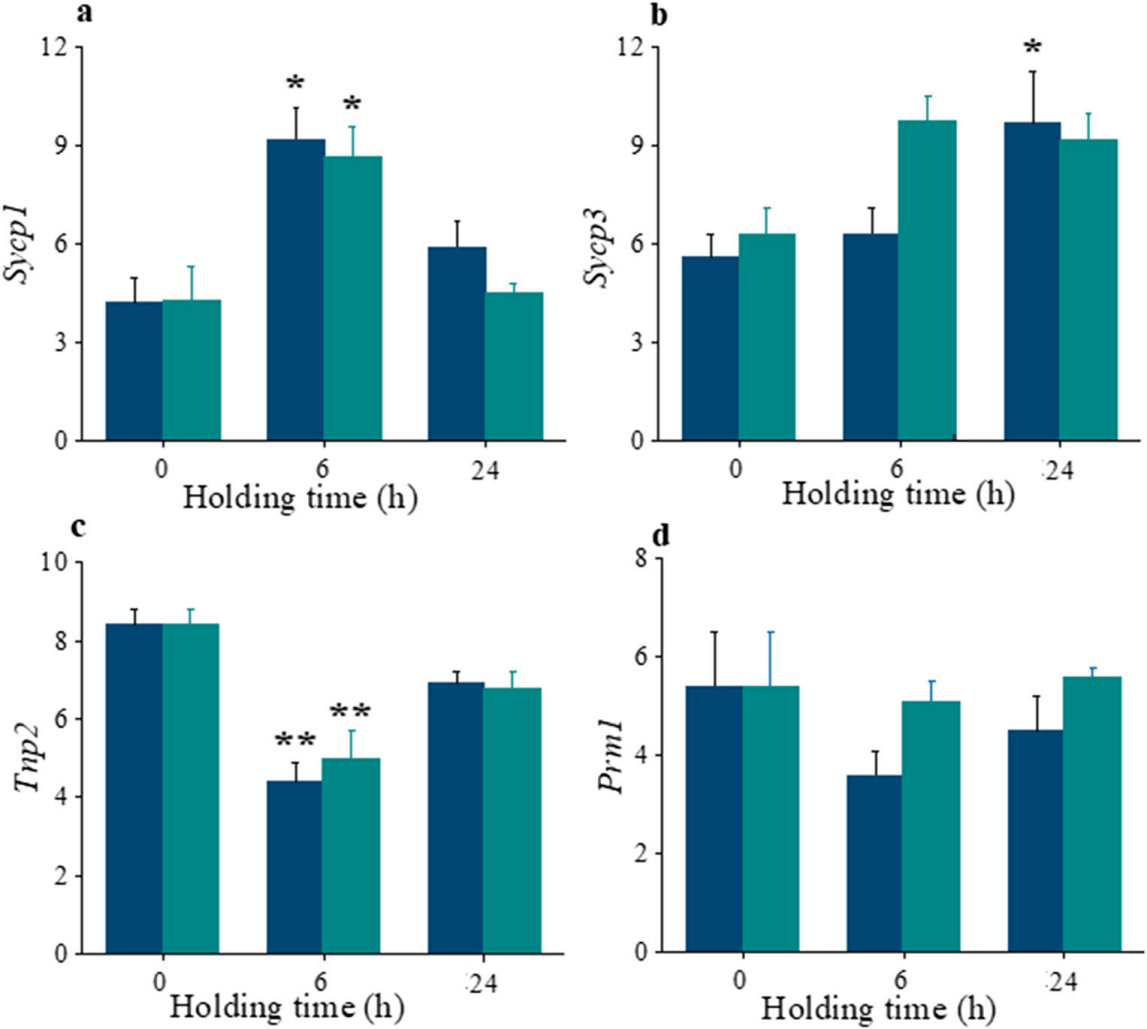
Gene expression analysis in ITT. Effect of varying holding time and temperature on the mRNA levels of **a** *Sycp1*, **b** *Sycp3*, **c** *Tnp2*, and **d** *Prm1* genes were assessed using qPCR. 6 dpp ITT, of 0 h interval cultured in vitro for 14 days were used as control in comparison to 6 and 24 h held ITT. The data is presented in fold change, ultra-profound-hypothermic (blue) and profound-hypothermic-temperature (teal), are normalized to 6 dpp testes (in vivo ITT) as to 1, with *Actb* and *Gapdh* used as housekeeping genes. ^*^P < 0.05 and ^**^P < 0.01 vs. 0 h (control), *N* = 3 trial

Synaptonemal complex proteins are meiosis specific proteins which play central role in synapsis, during leptonema phase of prophase I. Expression of two synaptonemal complex proteins *Sycp1* and *Sycp3* was assessed in cultured ITT subjected to ultraprofound-hypothermic and profound-hypothermic-temperature for varying time periods. The relative expression of transcripts was normalized to 6 dpp testes (in vivo ITT). The number of transcripts in 0 and 24 h groups was comparable between ultraprofound-hypothermic and profound-hypothermic-temperature groups of cultured ITT. However, the number of transcripts in 6 h group was significantly higher than 0 h (*P* < 0.05) (Fig. 6a). On the other hand, *Sycp3* levels did not vary significantly across the groups except between ultraprofound-hypothermic group at 24 h and corresponding group at 0 h (*P* < 0.05) (Fig. 6b). Though, ~ 1.5 folds change in *Sycp3* level was observed between 0 and 6 h cultured ITT in profound-hypothermic group, the differences were not statistically significant.

### Expression of Tnp2 and Prm1 Genes in ITT

Chromatin remodeling during spermiogenesis, results in the initial replacement of histones by transition proteins (*Tnp*), and then by protamine (*Prm*). *Tnp2* expression was studied in cultured ITT held at ultraprofound-hypothermic and profound-hypothermic-temperatures for varying time periods. Though, 0 h group did not show any difference in the number of transcripts between ultraprofound-hypothermic and profound-hypothermic-temperatures of in vitro cultured ITT, a significant decline (*P* < 0.01) in the number of transcripts was observed in both cultured ITT at 6 h holding time (Fig. 6c). There were no differences found between ultraprofound-hypothermic and profound-hypothermic temperatures of in vitro cultured ITT at 0 h (Fig. 6d). Though *Prm1* expression was lower in in vitro cultured ITT of ultraprofound-hypothermic group at 6 and 24 h, the differences were not significant.

## Discussion

Unlike most organs that mature during the fetal life, male gonads become fully functional only at puberty with the resumption of spermatogenesis. Prepubertal testes have unique architecture and niche hence, handling and manipulation of ITT at optimal conditions are important for cell growth and development that are required to develop effective therapeutic options for pediatric cancer survivors needing fertility restoration. Through analysis of subpopulations of testicular cells by flow cytometry, and organotypic culture system, this study evaluated the niche and quality of mouse ITT in response to holding at varying time periods and temperature prior to in vitro culture. No significant changes in cell viability, testosterone level, and in vitro proliferation ability were observed in ITT held at ultraprofound-hypothermic-temperature up to 24 h prior to the initiation of the culture. On the other hand, holding ITT at profound-hypothermic-temperature for 24 h significantly reduced the cell viability. Interestingly, after 14 days of organotypic culture, cell viability, and testosterone levels were comparable to the corresponding ultraprofound-hypothermic groups but with a reduction in the postmeiotic germ cell population. We believe that these insights will have potential translational value in the development of fertility restoration strategies for childhood cancer survivors.

Cryopreservation of ITT is a preferred method over isolated spermatogonial stem cells banking [24]. Handling of ITT prior to cryopreservation or in vitro culture may influence testicular architecture, endocrine function, and spermatogonial proliferation. Previous studies have provided substantial data to optimize ITT cryopreservation technique [14, 25–27]. Due to the unique architecture and constant turnover of stem cells, prepubertal testes may be more prone to impairments in functionality due to the way they are handled, transported, and maintained. Although attempts to achieve in vitro spermatogenesis using mouse and human ITT have been reported [7, 17, 18, 28–33], to our knowledge, this is the first report comparing the functionality of ITT handled at different temperature and time periods before the organ culture is initiated. We believe that this is an important prerequisite for the successful in vitro generation of spermatozoa.

Temperature can fluctuate while shipping the testicular biopsy sample and during the holding of the tissue. Hence, we used ultraprofound-hypothermic-temperature (~ 4 °C) in which ITT is usually transported and maintained prior to the storage. Profound-hypothermic-temperature (room temperature, ~ 22 °C) mimics tissue handling outside the ultraprofound-hypothermic-temperature or when time taken for shipment is long. Cell viability did not change significantly until 6 h of holding at ultraprofound and/or profound-hypothermic-temperature whereas a marginal loss in viability was evident at the end of 24 h, in profound-hypothermic group. This raises concern about the quality of ITT when shipment to the banking facility is delayed. Earlier study in human ITT showed no change in the structural integrity up to 3 days of holding at 4 °C [13]. However, in our study, after 14 days of in vitro culture, the cell viability, testosterone production, and the number of postmeiotic population were identical across both ultraprofound and profound-hypothermic-temperature groups and at both 6 h holding, but significantly reduced in 24 h holding at profound-hypothermic-temperature. This emphasizes the fact that the loss of viability does not impair the in vitro culture outcome, in terms of cell viability or proliferation of germ cells but impacts the somatic cell function. Importantly, ITT subjected to profound-hypothermic-temperature for 6 h had significantly lower number of postmeiotic population in comparison to 0 h postorganotypic culture.

During the meiotic prophase I, synapsis of homologous chromosomes is achieved by the formation of a large zipper-shaped protein complex known as the synaptonemal complex [34]. SYCP2 and SYCP3 proteins initiate the formation of fibrous cores alongside the homologous chromosomes [35, 36]. Moreover, in the absence of *Sycp1* (*Sycp1−/−*) in spermatocytes, the homologous chromosomes do not synapse, causing the cells to undergo apoptosis at pachytene stage [37]. In the current study, the levels of *Sycp1* and *Sycp3* transcripts were higher at 6 and 24 h respectively, in ultraprofound-hypothermic and profound-hypothermic-temperatures. The elevated levels of *Sycp* transcripts could be due to the significantly higher S-phase population in the group, resulting in delayed progression to postmeiotic population compared to 0 h post organotypic culture. The prolonged/delayed S-phase could be the time taken for cell population to repair the DNA damage before duplication. The meiotic phase of spermatogenesis is followed by elongation phase of spermiogenesis, which involves elongating and condensing spermatids undergoing extensive chromatin remodeling, where the histones are first replaced by transition proteins (TP), and in turn replaced by protamines (PRM) [38, 39]. The decreased levels of *Tnp2* in 6 h profound-hypothermic group could account for a significantly lower 1C population in the group. However, the nonsignificantly different *Prm1* transcripts among all the groups and intervals studied might be an indication of nuclear condensation in the spermatids.

On the other hand, even up to 6 h exposure to warm-ischemic-temperature before the organotypic culture resulted in a significant impairment in the cell viability and testosterone levels. Warm ischemia is known to have adverse effects on mammalian spermatogenesis leading to increased apoptosis or DNA damage in germ cells [40, 41]. Handling ITT at mild-warm-ischemic condition for a period of 24 h in the present study resulted in a significant loss of viability before and after the in vitro culture. Stem cell population exposed to hyperthermic temperature (> 37 °C) can inhibit self-renewal through S-phase cell cycle arrest [42]. Interestingly, the number of S-phase population in our study was significantly higher in both profound-hypothermic and ultraprofound-hypothermic groups after 6 h of holding time. At this juncture, we cannot explain the biological significance of this observation. Nonetheless, ITT are not normally exposed to this temperature except in case of improper handling during transportation, especially in low resource settings hence, we have not performed organotypic culture from this group.

Studies using several experimental models have shown that holding and manipulation at 4 °C is optimal for ITT [10, 14, 43, 44] and human ITT can have intact structure up to 3 days at 4 °C [13]. It has been shown that storage of human ITT at 37 °C but not at 4 °C or room temperature caused a significant increase in the number of apoptotic cells compared with fresh control [12] which is in agreement with our results where mild-warm-ischemic temperature also resulted in increased cell death. Earlier studies have used either cell morphology analysis [12] or xenografting [44] as markers to assess the functional competence of ITT. However, our study is unique in a way that holding at different time periods was tested to address the functionality and cell niche by organ culture method. Our results have shown that holding at profound-hypothermic-temperature up to 6 h can have minimum detrimental effects as evidenced by the viability assay and cell proliferation analysis, postorganotypic culture.

The main limitation of our study is that only fresh ITT were tested without cryopreservation hence, does not mimic the human clinical situations. However, it is convincing to note that fresh and cryopreserved prepubertal testicular fragments have comparable functionality in relation to spermatogonial survival and proliferation, intratubular cell apoptosis, Sertoli cell maturation and proliferation, and testosterone production by Leydig cells [45]. Similarly, DNA methylation and histone methylation and acetylation were comparable between fresh and cryopreserved mouse ITT [46]. Though we have assessed the function of Leydig cells within ITT subjected to different holding conditions by their ability to secrete testosterone, Sertoli cell viability, and functional ability of in vitro derived spermatozoa were not assessed. Hence, the tested handling conditions having an influence on the viability and functional ability of other cell types and spermatozoa warrants further investigation. It is important to note that spermatogenic potential in vitro is dependent on specialized sites in the genome and therefore the organ culture conditions are suboptimal for some strains of mice [45]. Due to ethical restrictions in using human ITT, mouse model still serves as an important tool to address the technical questions in developing strategies for fertility restoration.

In conclusion, we report that holding ITT at ultraprofound-hypothermic-temperature is the most suitable condition for organotypic culture. Though, cell viability and endocrine function are not affected by short-term exposure of ITT at profound-hypothermic-temperature, impaired germ cell proliferation may compromise the number of postmeiotic germ cells derived from in vitro culture. We believe that the data derived from our experiments albeit in mouse model will be of immense value in future prepubertal fertility restoration research.

## Supplementary Information

Supplementary Figure 1 (PNG 848 kb)

High Resolution Image (TIF 169 kb)

Supplementary Figure 2 (PNG 4620 kb)

High Resolution Image (TIF 1122 kb)

Supplementary Table 1 (DOCX 13 kb)

## Data Availability

The data and material that support the findings of this study are available from the corresponding author upon request.
