# Supplementary figures and images for: Impact of Temperature and Time Interval Prior to Immature Testicular-Tissue Organotypic Culture on Cellular Niche
Source: Reprod Sci. 2020 Dec 15;28(8):2161–73. doi: 10.1007/s43032-020-00396-z (PMC8289760; doi:10.1007/s43032-020-00396-z)

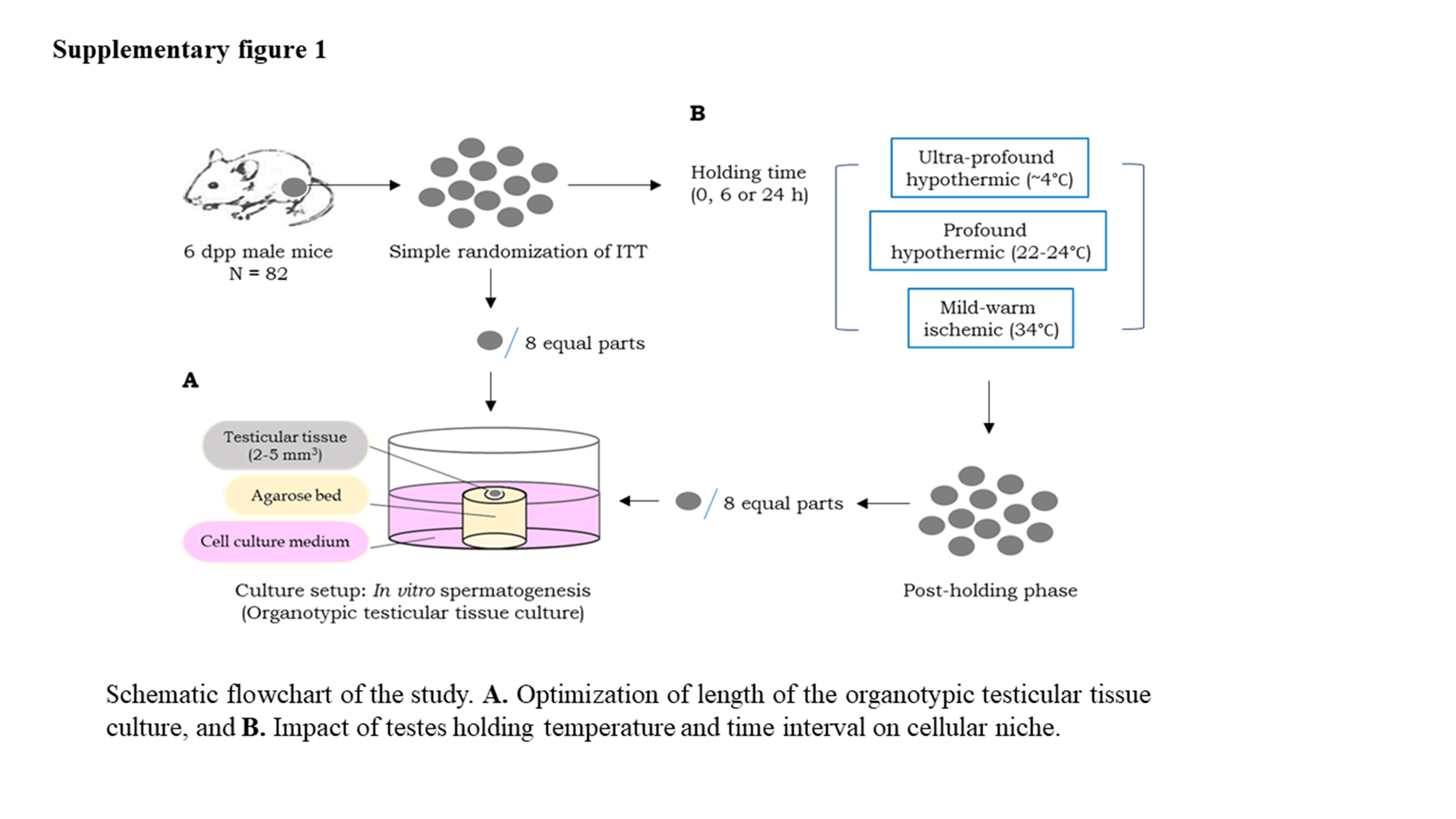

Supplement: Supplementary file 1 — (PNG 848 kb) [file 43032_2020_396_Fig7_ESM.png]

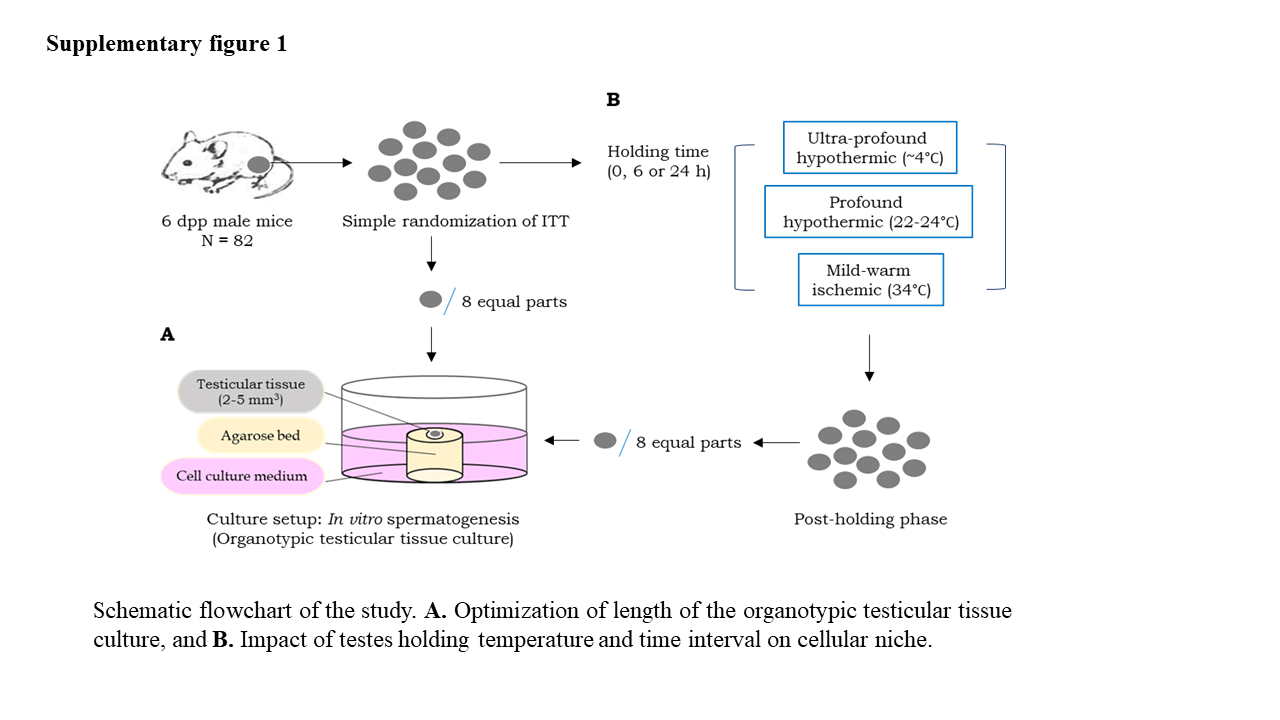

Supplement: Supplementary file 2 — High Resolution Image (TIF 169 kb) [file 43032_2020_396_MOESM1_ESM.tif]

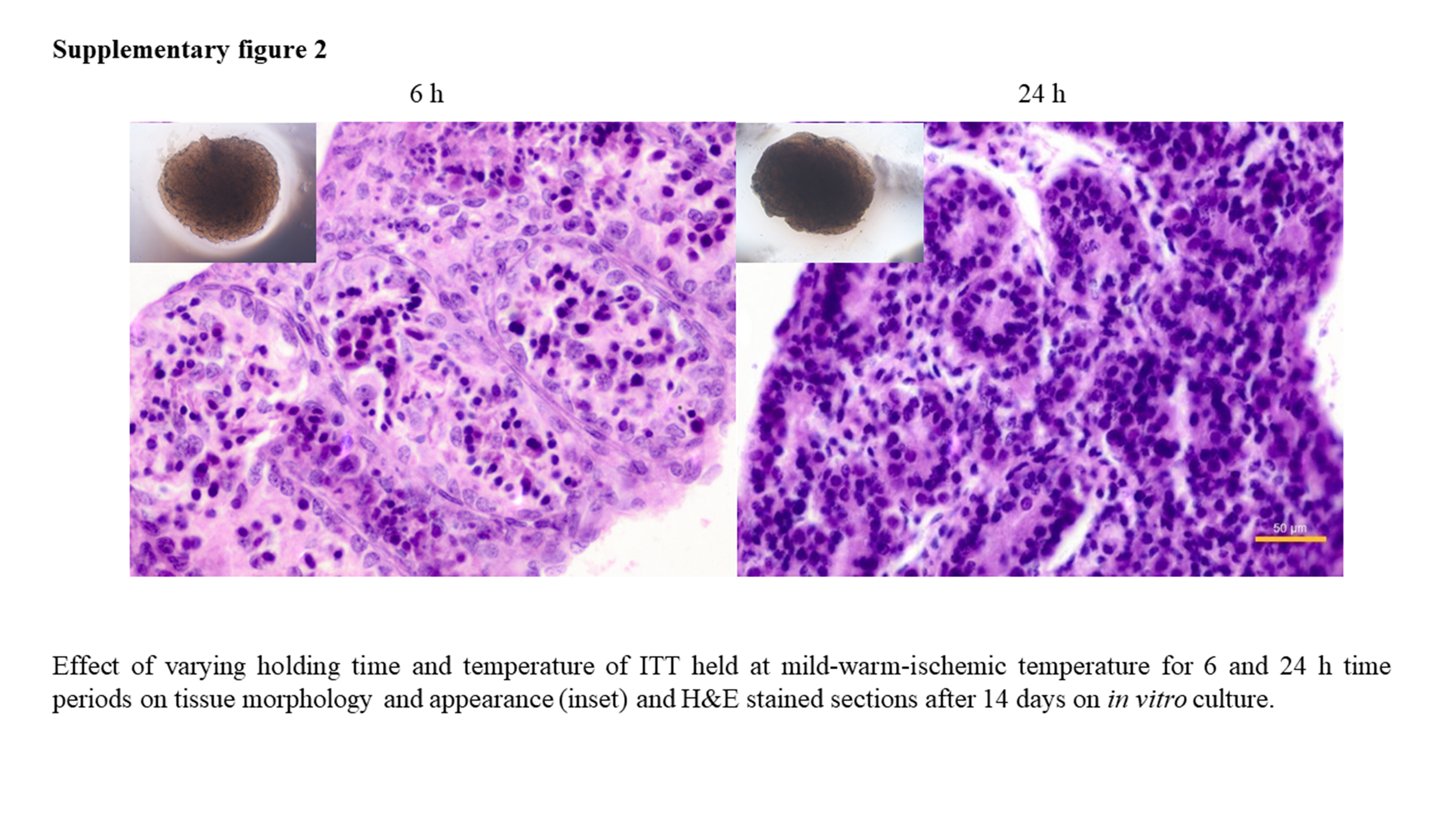

Supplement: Supplementary file 3 — (PNG 4620 kb) [file 43032_2020_396_Fig8_ESM.png]

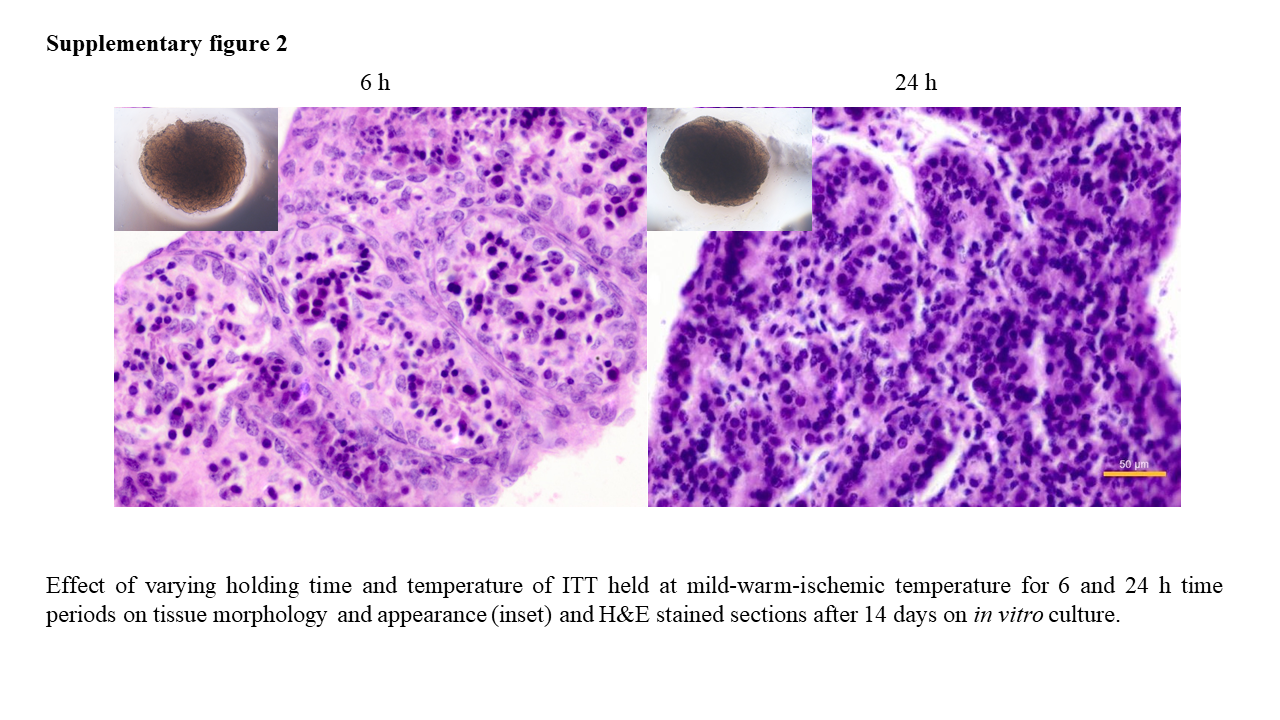

Supplement: Supplementary file 4 — High Resolution Image (TIF 1122 kb) [file 43032_2020_396_MOESM2_ESM.tif]
