# Supplementary material for: Impact of Temperature and Time Interval Prior to Immature Testicular-Tissue Organotypic Culture on Cellular Niche
Source: Reprod Sci. 2020 Dec 15;28(8):2161–73. doi: 10.1007/s43032-020-00396-z (PMC8289760; doi:10.1007/s43032-020-00396-z)
Supplement: Supplementary file 5 — (DOCX 13 kb) [file 43032_2020_396_MOESM3_ESM.docx]

| List of Taqman assays used for the study | |
| --- | --- |
| Gene | Taqman assay gene reference |
| *β-Actin* | Mm00607939_s1 |
| *Gapdh* | Mm99999915_g1 |
| *Sycp1* | Mm01297993_m1 |
| *Sycp3* | Mm00488519_m1 |
| *Tnp2* | Mm00726979_s1 |
| *Prm1* | Mm01342731_g1 |

**Supplementary Table 1:** Taqman probes for quantitative polymerase chain reaction (qPCR)
